# Rapid development of a transferable Raman model using high‐throughput cell culture for monitoring monoclonal antibody titer

**DOI:** 10.1002/btpr.88506

**Published:** 2026-04-05

**Authors:** Alexandra Umprecht, Nicholas Uth, Haenah Kim, Oliver Spadiut, Yang Yang

**Affiliations:** ^1^ Pharmaceutical Sciences, R&D Baxalta Innovations GmbH, a member of the Takeda group of companies Vienna Austria; ^2^ Institute of Chemical Environmental and Bioscience Engineering, TU Wien Vienna Austria; ^3^ Pharmaceutical Sciences, R&D Takeda Pharmaceutical Company Limited Lexington Massachusetts USA; ^4^ Chemical Engineering University of Massachusetts Lowell Lowell Massachusetts USA

**Keywords:** high‐throughput upstream development, model transferability, Raman spectroscopy

## Abstract

Monoclonal antibody (mAb) titer monitoring is a key capability during process development and optimization, enabling timely decision making and increasing the speed of development. Raman spectroscopy is a prominent process analytical technology (PAT), but resource‐efficient calibration strategies for the development of transferable models are limited. This work demonstrates the development and successful transfer of a calibration model for monoclonal antibody concentration between two different cell lines with varied metabolic profiles expressing different antibodies. The root mean square error of prediction (RMSEP) for titer in the source cell line (0.266 g L^−1^) was comparable to that of the target cell line (0.325 g L^−1^). The transferable model was achieved by conducting a spiking study in a high‐throughput parallel bioreactor system. Different experimental approaches with models trained on spiked versus native samples were compared. This analysis revealed that model transferability was influenced by the degree of correlation of lactate with antibody titer in the source process, emphasizing the importance of process knowledge in the development of Raman calibration models. Overall, the study presents evidence of the feasibility of transferable titer models, marking a significant advancement in process monitoring capabilities for high‐throughput cell culture as well as introducing a generic methodology for calibration of transferable models in the context of upstream bioprocessing.

**Abbreviations**
CHO: Chinese hamster ovary
HPLC: high‐performance liquid chromatography
iVCD: inoculation viable cell density
LV: latent variable
mAb: monoclonal antibody
MPC: model‐predictive control
PAT: process analytical technology
PLS: partial least squares
RMSE: root mean square error
RMSECV: root mean square error of cross‐validation
RMSEP: root mean square error of prediction
RS: Raman spectroscopy
TCD: total cell density
VCD: viable cell density
VIP: variable importance in projection

## 1 INTRODUCTION

In recent years, the biopharma industry has been prioritizing the addition of new process analytical technologies (PAT) to their cell culture platforms.^1^ The adoption of PAT has accelerated in recent years with FDA guidance in 2004 and the emergence of numerous diverse approaches.^2^, ^3^ Raman spectroscopy (RS) is a prominent PAT tool due to its appealing properties for bioprocesses: compatibility with aqueous systems, high selectivity to molecular structure, and the ability to simultaneously measure multiple analytes.^4^ Since the early 2010s,^5^ RS has been used extensively to perform in‐process measurements of key analytes (e.g., glucose, lactate, glutamine, glutamate and other amino acids, ammonium), cellular attributes (e.g., cell density and/or viability), as well as protein quality attributes (e.g., titer,^4^ glycosylation^6^, ^7^). Most applications in the literature focus on monitoring, but applications in model‐predictive control (MPC),^8^, ^9^ are also becoming more common.

### 1.1 Developing specific and transferable models for monoclonal antibody titer

Titer monitoring is of special interest, as this can be considered the main performance indicator of cell culture processes. Offline methods for antibody titer such as high‐performance liquid chromatography (HPLC) are often performed in dedicated analytical labs, which can lead to an increased lead time and delayed decision making. RS is typically implemented as either an in‐line method (via a probe placed within the bioreactor) or an at‐line method (e.g. a liquid handler sampling to a flow cell for high‐throughput bioreactors), but in each scenario it can provide titer values within minutes. The first publication demonstrating monitoring of titer in‐line by RS was by André et al. in 2015.^10^ Subsequent studies addressed critical challenges for successful titer model implementation. Santos et al. investigated titer model selectivity (i.e., whether the model included spectral features that could be assigned to the Raman signal of an antibody), suggesting low selectivity of the investigated model.^11^ Webster et al. explored generic models (across cell lines), which were feasible for analytes such as glucose and lactate but not for antibody titer, possibly due to correlations of titer to viable cell density (VCD) and culture time.^12^ The first successful generic titer model was published by Yilmaz et al. for perfusion cell culture.^13^ However, the authors do not discuss the degree to which the metabolic profiles of the investigated cell lines varied, which complicates the assessment of model transferability beyond the IgG subclasses. More recently, Machleid et al. showed clone‐related bias of titer models, potentially as a result of correlations with other cell culture components such as lactate, glutamate and glutamine.^14^ These works highlight the two important and often related desirable properties of Raman models—selectivity and transferability.

Achieving a model which is highly selective for a given analyte is key to successful Raman implementation. While RS is highly selective to molecular structure, the typical cell culture consists of numerous components which may have overlapping spectroscopic signals.^15^ A common chemometric approach is to use partial least squares (PLS) regression to train a model for a given analyte. The selectivity of a PLS model depends on the absence of strong multivariate correlations in the training dataset, which is often difficult to achieve using native cell culture samples. If the PLS model (source domain) is trained on a dataset with correlations that are absent from future datasets (target domain), systematic errors will be observed when the model is applied to the target domain. Lack of model selectivity can be assessed by investigating β‐coefficients^16^ or variable importance in projection (VIP) scores^11^ of the PLS regression model and assigning them to the expected pure analyte peaks.

A closely related challenge in implementing Raman models is their transferability. Developing generalized models that can be applied across different cell lines, products, processes, scales and spectral hardware is highly desirable, as recalibrating models for every process change is very resource intensive. There have been different levels of success in achieving transferable models based on the analyte as well as type of transfer task (i.e. encountered change in the data). Transferable models across scales (small scale—pilot—manufacturing) have been developed for glucose, where robust models (normalized RMSEP <10%) could be calibrated without addition of the target manufacturing scale data, but the same was not true for VCD models.^17^ Transfer across cell lines and scales (5 L to 10 L) was demonstrated by Webster et al. for glucose, lactate, ammonium, VCD and TCD, but achieving satisfactory performance was not possible for glutamate and product titer.^12^ Similarly, Mehdizadeh et al. showed that generic models with acceptable performance could be achieved for glucose, lactate and VCD when changing between scales (5 L to 500 L scale) or cell lines.^18^ However, for certain scales, cell line and analyte combinations, relatively high errors were observed. Kozma et al. demonstrated deploying a glucose model calibrated using shake‐flasks to 10 L and 100 L scales.^19^ Recently, Wan et al. showed transfer of glucose, lactate, glutamine, glutamate and titer model across various scales and flowrates in a perfusion process.^20^ While the previous studies used CHO cell lines, André et al. included Sf9, HeLa and HEK‐293 cell lines. As long as some data from a given cell line were included in the training set, it was possible to achieve satisfactory predictions for glucose and lactate, which otherwise showed high prediction bias.^21^

Common solutions for generating robust, specific and transferable models are to generate very large and/or highly varied datasets from multiple processes and scales,^12^, ^17^, ^18^, ^21^ incorporate spiking studies into the data sets^11^, ^18^ or use chemometric methods, such as variable selection,^22^ pre‐processing^23^ or calibration transfer and maintenance methods.^24^ However, challenges arise from each of these. The first two approaches require significant amounts of time, labor, and materials, often prohibitive during standard process development timelines. The third approach often requires data from the target process; however, such data is typically unavailable during the given process development phase. Additionally, chemometric approaches often require specialized methods, which are commonly not available in commercial software.^25^

### 1.2 High‐throughput approaches for model development

Modern bioprocess development increasingly utilizes high‐throughput miniaturized bioreactor systems, which increase the speed of development through parallelization. Such systems also enable the generation of large data sets for Raman model calibration when coupled to a Raman spectrometer. One potential configuration is sampling the cell culture into microtiter plates (e.g. 96‐well) and using an at‐line or offline high‐throughput Raman microscope to record the spectra.^16^, ^26^, ^27^ A significant advancement in high‐throughput calibration dataset generation was achieved through the integration of Raman spectrometers with commercially available ambr® bioreactors.^14^, ^28^, ^29^, ^30^ In this set‐up, samples are delivered from each vessel to an integrated flow cell via the built‐in liquid handler. Such automation boosts efficiency as it requires less manual intervention, increases consistency of at‐line data capture and reduces human error. The increased bioreactor count per operator per run also allows for more conditions to be executed (incl. platform and non‐platform processes set‐points, duplicates and controls), ultimately leading to increased model robustness with respect to process changes. Finally, automation also significantly simplifies spiking the native cell culture with target analytes. An important limitation of high‐throughput cell culture systems is the reliance on at‐line sampling to acquire spectra, which necessitates model transfer when applied to scales which use in‐line probes to record Raman spectra.

The performance of models calibrated with a Raman microscope or an at‐line flow cell must be validated using in‐line probes. Such transfer was explored by Rowland‐Jones et al. who investigated the transfer of a glucose model calibrated using predominantly ambr15® data (with a single run at 50 L scale) and using it to predict a 50 L scale equipped with a spectral port. However, relatively high errors of 0.99 g L^−1^ were observed, even following one‐point recalibration.^29^ Improved transfer was reported by Classen et al. (not peer‐reviewed),^31^ demonstrating a model calibrated at the ambr®250 scale using an at‐line flow‐cell successfully transferred to a 10 L scale with an in‐line probe for glucose and lactate models. This highlights the feasibility of such transfers, though further studies assessing various probe, flow cell, and spectrometer combinations are needed.

### 1.3 Study aims

In the present study, we developed a predictive Raman‐based model for monoclonal antibody titer, which is transferable between cell lines with varied metabolic profiles producing two different monoclonal antibodies. The calibration dataset for the model was generated using automated high‐throughput cell culture with an integrated Raman spectrometer, enabling the creation of a large native dataset, which was further expanded by spiking of a purified antibody. Use of automated high‐throughput instrumentation resulted in model development with expedited timelines and reduced manual operations and material usage. Furthermore, chemometric analysis of the resulting models revealed the root causes of lack of transferability, bringing additional insights into the importance of process knowledge for the development of calibration models. Overall, this study demonstrates a transferable titer model and highlights the role of high‐throughput scale‐down approaches in the calibration of Raman models.

## 2 MATERIALS AND METHODS

### 2.1 Cell culture and data collection

Two in‐house generated Chinese hamster ovary without glutamine synthetase (GS‐CHO) cell lines expressing two distinct monoclonal antibodies were used in the experiment and referred to as cell line A and cell line B. Both cell lines were cultured in proprietary media in a fed‐batch mode. The cell lines were initially expanded in shake flasks (250 mL and 1 L) from frozen vials and subsequently used to inoculate an ambr®250 parallel high‐throughput bioreactor equipped with 24 vessels (Sartorius AG, Göttingen, Germany). The experiment was run utilizing 250 mL filtered bioreactors (with a working volume of 200 mL) without baffles. The individual vessels differed in inoculation density (iVCD), culture temperature, and volume of feed; the overview is provided in Table 1. In general, the cultures were run at 37°C with daily feeding done via automatic bolus addition. Cell culture conditions were selected to affect growth kinetics and reduce time‐correlation in the training data. Four runs (referred to as R1A‐R4A) were run for cell line A and two (R1B, R2B) runs were executed for cell line B. All experimental runs lasted for 14 days.

**TABLE 1:** Overview of conditions, runs and number of vessels for each cell line.

Cell Line	No. of runs	Condition	No. of Vessels	Comment
A	4	Control	16	Target iVCD = 3.5 × 10^5^ cells / mL
		High iVCD	2	Target iVCD = 1 × 10^6^ cells /mL
		Low iVCD	11	Target iVCD = 2 × 10^5^ cells / mL
		Media exchange	3	One media exchange performed
		Low feed	5	Feed at 50% concentration of control
		Low temp	5	Inoculation temperature at 35°C and shifted up on day 4
		Low feed + low temp	2	Combination of above two
Total:			44	
B	2	Control	6	Target iVCD = 3.5 × 10^5^ cells / mL
		High iVCD	8	Target iVCD = 1 × 10^6^ cells /mL
		Low iVCD	8	Target iVCD = 2 × 10^5^ cells / mL
		Low feed + low temp	8	Combination of both low feed and low temp start
Total:			30	

The ambr®250 was coupled with an at‐line analyzer (BioProfile® Flex2, Nova Biomedical, Wilmington, MA) and a Raman spectrometer (HyperFlux™ Raman Analyzer, Tornado, Mississauga, ON Canada) via the BioPAT®Spectra module as previously described (see Figure 1 in Graf et al. for a drawing of the set‐up).^28^ The software of ambr®250 was used to control both the at‐line analyzer and the spectrometer.

**FIGURE 1:**
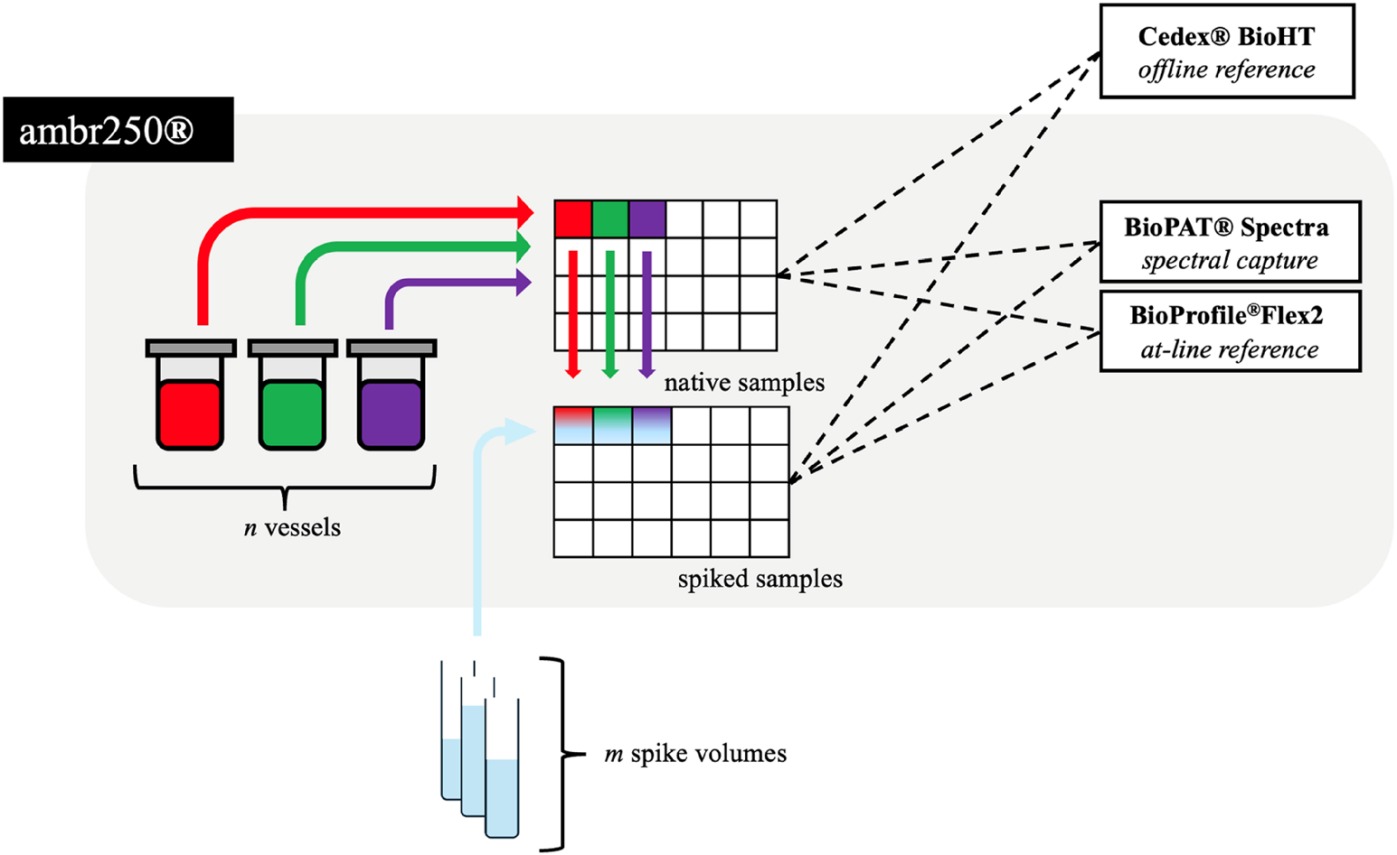
Illustration of sampling methodology. A sample of the native cell culture was drawn from each of *n* vessels once a day and deposited into a sampling cup. For each of these samples, aliquots were removed and used for spectral measurement, at‐line and offline analytics and for generation of spiked samples. For each spiked sample, the spectra and offline/at‐line analytics were recorded. Shaded regions show operations which were automated and performed by the liquid handler of ambr®250. Operations outside the shaded regions (spike solutions preparation, offline analytics) were performed manually.

A cell culture sample was drawn from each vessel once daily using the ambr®250 liquid handler. This sample was further subsampled and used for at‐line, offline and spectral measurement as well as for the generation of a spiked sample (Figure 1). Daily at‐line measurements of viable cell density, viability, glucose, lactate, glutamate, glutamine, and ammonia were performed using BioProfile® Flex2. Offline measurements of IgG concentration were performed using a Cedex® BioHT analyzer (Roche Custom Biotech, Penzberg, Germany) starting from day 3 (no measurable mAb production was assumed to take place prior to day 3) to the end of culture.

Raman spectra were recorded at 785 nm with a laser power of 495 mW. Exposure time and the number of accumulations were determined using a reference run to achieve approximately 70% of the maximum linear range of the detector as per manufacturer's instructions. To achieve the desired percentage of the detector range, the acquisition time was changed as the cell culture progressed (ranging from 2800 to 1800 ms) in order to account for the increasing signal intensity in the course of the run (due to increasing autofluorescence^32^). A total of 30 accumulations were taken for each spectral capture (single sample within the BioPAT®Spectra flow cell without active flow/mixing) and averaged. Additionally, ad hoc spectra were recorded by manually delivering sample to the BioPAT®Spectra module to generate reference spectra of non‐cell culture samples (e.g. purified mAb used for spiking). Samples for some conditions/days were not successfully collected due to flow cell filling error, thus removed during initial rounds of data cleaning, see Section 2.2.1.

Spiking was performed utilizing a combination of automated steps via the ambr®250 and manual pipetting (Figure 1). A spiking plate consisting of a metal assay tray filled with 24 BioProfile® Flex2 cups was prepared for the experiment and pre‐loaded manually with purified mAb (purified using affinity chromatography with protein A) from each cell line (produced using a separate bench‐scale run at control conditions). The amount pipetted into each well was randomized. The purified mAb consisted of a proprietary protein A elution buffer and was concentrated prior to use. Using the ambr®250 liquid handler, 300 μL of bioreactor sample was deposited into an empty cup with a given concentration of mAb and mixed. The spiked sample was deposited to the BioPAT® Spectra module and spectral capture was recorded. Each spiked sample was additionally measured offline on the Cedex® BioHT analyzer.

### 2.2 Spectral model development

The modeling workflow included dataset cleaning and outlier removal, dataset split, pre‐processing, and model complexity selection based on cross‐validation and model testing on an independent test set.

#### 2.2.1 Dataset cleaning, outlier removal, dataset split

Data points with incomplete flow cell filling were identified by the missing prominent water peak using PCA analysis (spectra affected by dripping would deviate from unimpacted spectra across the second component, Figure S1) and were removed. Outlier at‐line / offline measurements were removed e.g. where values of the analyte were unexpectedly low, suggesting an error in the at‐line / offline method (representative example in Figure S2). The dataset was then split into a training set (cell line A) and two test sets (cell line A, cell line B) such that data from all vessels which belonged to R1A, R3A and R4A were designated as the training set and R2A vessels as the test set for cell line A. Both cell line B runs were part of the test set as cell line B was not involved in model training. The assignment of runs into the training and test sets was arbitrary.

#### 2.2.2 Preprocessing optimization and model selection

Chemometric modeling was performed using Python utilizing NumPy,^33^ scikit‐learn,^34^ pandas,^35^ and matplotlib packages.^36^ Following the dataset split, five‐fold grouped (by vessel) cross‐validation was performed using data for cell line A to identify optimal pre‐processing and optimal model complexity (number of latent variables (LVs)). The overall pre‐processing approach was based on Engel et al.,^37^ with pre‐processing grouped into three blocks, where one, two or three functions (one from each block) may be applied to generate the pool of pre‐processed arrays. The order of the blocks was driven by recommendations of Rinnan et al.,^38^ so that scatter correction is applied first, followed by baseline correction and denoising. Total number of pre‐processing combinations was 1040 and the individual filters used are shown in Table 2 with parameters used for each pre‐processing function specified in Table S1. Unless stated otherwise, functions used were of the same form as described in the nippy Python library.^39^ Baseline removal was performed using the pybaselines library.^40^ References to original research for the above‐mentioned pre‐processing approaches can be found in the documentation of the respective libraries.

**TABLE 2:** Pre‐processing filters used during pre‐processing optimization.

Block 1	Block 2	Block 3
Scatter correction	Baseline correction	Smoothing
Standard Normal Variate (SNV) (1)	Modified Polynomial (3)	Savitzky–Golay (no derivative) (4)
Robust Standard Normal Variate (RSNV) (3)	Improved Modified Polynomial (3)	Asymmetric Least Squares (*λ* <10^2^) (6)
	Asymmetric Least Squares (AsLS) (*λ* >10^5^) (4)	
	Adaptive Iteratively Reweighted Penalized Least Squares (3) (airPLS)	
	Adaptive MinMax (1)	
	Savitzky–Golay (S‐G) (with derivative) (12)	
Total: 4	Total: 26	Total: 10

For each pre‐processing, the optimal number of LVs was identified by using a *t*‐test for significant difference (*p* = 0.05) in residuals for models with different numbers of LVs (up to 10 LVs).^41^ A given number of LVs was selected as optimal where there was no significant difference in residuals between the tested model and the model with minimal root mean square error of cross‐validation (RMSECV).^23^, ^41^ The procedure was repeated for each analyte. A spectral range of 600–1700 cm^−1^ was used for all models. Using the identified optimal model, predictions were generated for the test set of cell‐line A (R2A) and cell line B (R1B and R2B). Model performance in cross‐validation and test was performed by calculating RMSE, normalized RMSE (nRMSE, normalized by range) as well as slope, bias and *R* ^2^. The selectivity of the model was determined by comparing the boot‐strapped *β*‐coefficients (re‐sampled training set for both the native and spiked model) to the spectra of mAbs from cell line A and B.

## 3 RESULTS

### 3.1 Cell line comparison

**FIGURE 2:**
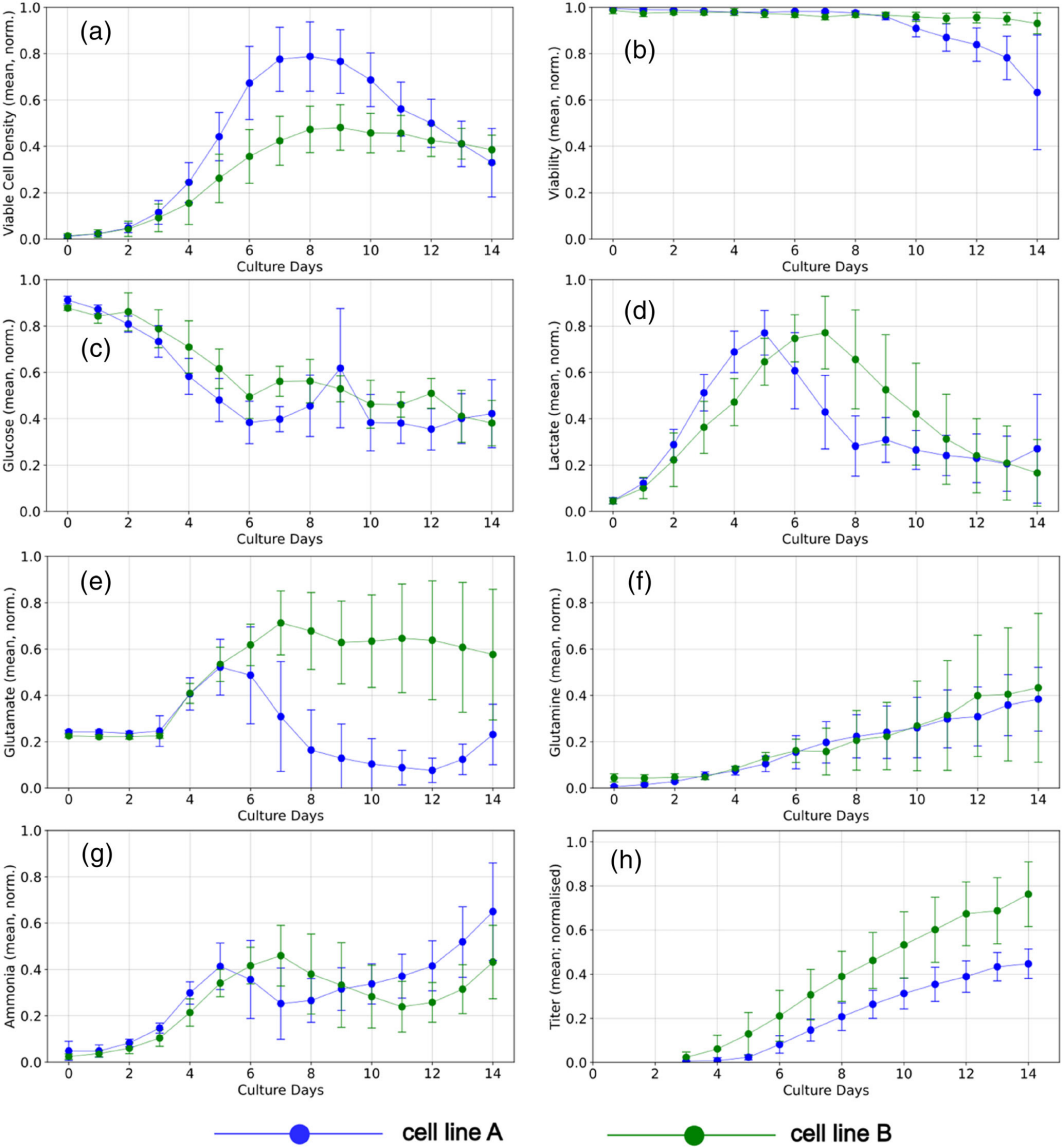
Comparison of cell culture performance of cell line A and cell line B. Normalized mean values of at‐line and offline measurements across all vessels with standard deviation for viable cell density (a), viability (b), glucose (c), lactate (d), glutamate (e), glutamine (f), ammonia (g) and protein titer (h) are shown for cell line A (in blue) and cell line B (in green). Day 0 refers to measurements at inoculation. Titer data were recorded only from day 3 onwards.

Cell line performance across the two cell lines was assessed by calculating mean values and standard deviations acquired using at‐line and offline analytics across all vessels for the given cell line. Cell line A showed higher mean viable cell density (Figure 2a) compared to cell line B but showed lower mean viability (Figure 2b) towards the end of the run (decreasing from day 8 onwards). Both cell line A and cell line B used glucose as a carbon source and showed a similar glucose profile (Figure 2c) with differences in concentration increases caused due to the Raman sampling protocol and the proximity of sampling time to the time of feeding. The two cell lines differed in the timing of the metabolic shift, where the cells changed from lactate production to lactate consumption,^42^ which took place approximately day 5 for cell line A and day 7 for cell line B (Figure 2d). Glutamate concentration (Figure 2e) increased in both cell lines during the run as a result of feeding and decreased as cells consumed glutamate as a carbon source, with concentrations of glutamate in cell line A being lower due to higher viable cell density. Glutamine (Figure 2f) concentration steadily increased in both cell lines as a byproduct of the CHO metabolism. Ammonium (Figure 2g) increased throughout the run in both cell lines, though the rate of ammonium production seems to correlate with the occurrence of the lactate switch. Finally, protein titer (Figure 2h) was lower in cell line A compared to cell line B (measured only from day 3 onwards, as prior to day 3 no measurable protein production is assumed). Overall, the two cell lines showed diverse profiles in terms of growth, viability, and metabolism; therefore, were deemed a good fit to study model transferability.

### 3.2 Pre‐processing optimization and spectral analysis of native and spiked spectra

In the first stage of model calibration for cell line A, a pre‐processing optimization was performed by screening a library of pre‐processing filters while optimizing the model complexity (number of LVs) for glucose, lactate, and antibody titer (with and without spiked samples). Selected optimal pre‐processing for each analyte is shown in Table 3. While the objective of the study was the calibration of a transferable model for antibody titer, glucose and lactate models were also assessed and served as a transferability benchmark, since successful transfer has been previously described.^12^

**TABLE 3:** Model training and model testing parameters for Glucose, Lactate, and Titer models.

Model training (Cell line A)				
Analyte	Glucose (native)	Lactate (native)	Titer (native)	Titer (spiked)
No. of Samples (*n*)	440	451	275	381
Pre‐processing	SNV; iModPoly; S‐G	SNV; AsLS; S‐G	SNV; S‐G (1st der); AsLS	SNV; AsLS; S‐G
LVs	8	6	6	7
(n)RMSECV (g L^−1^; %)	0.126; 1.618	0.064; 1.976	0.127; 7.759	0.291; 6.570
Range (g L^−1^)	0.090–7.860	0.050–3.290	0.081–1.721	0.081–4.505
*R* ^2^	0.995	0.994	0.932	0.905
Bias (g L^−1^)	0.020	0.010	0.052	0.116
Slope	0.995	0.992	0.932	0.912

Model testing (Cell line A)				
Analyte	Glucose (native)	Lactate (native)	Titer (native)	Titer (spiked)
No. of Samples (*n*)	93	93	60	81
(n)RMSEP (g L^−1^; %)	0.158; 3.002	0.087; 2.808	0.125; 7.221	0.266; 7.979
Range (g L^−1^)	2.010–7.370	0.150–3.260	0.091–1.818	0.091–3.426
*R* ^2^	0.992	0.992	0.953	0.894
Bias (g L^−1^)	−0.086	0.031	0.123	0.176
Slope	1.022	1.010	0.873	0.882

Model testing (Cell line B)				
Analyte	Glucose (native)	Lactate (native)	Titer (native)	Titer (spiked)
No. of Samples (n)	397	406	290	370
(n)RMSEP (g L^−1^; %)	0.274; 4.735	0.118; 3.322	0.776; 23.934	0.325; 7.976
Range (g L^−1^)	1.980–7.761	0.060–3.610	0.055–3.297	0.055–4.134
*R* ^2^	0.985	0.989	0.883	0.904
Bias (g L^−1^)	−0.413	−0.005	−0.230	−0.125
Slope	1.107	1.034	0.667	1.031

**FIGURE 3:**
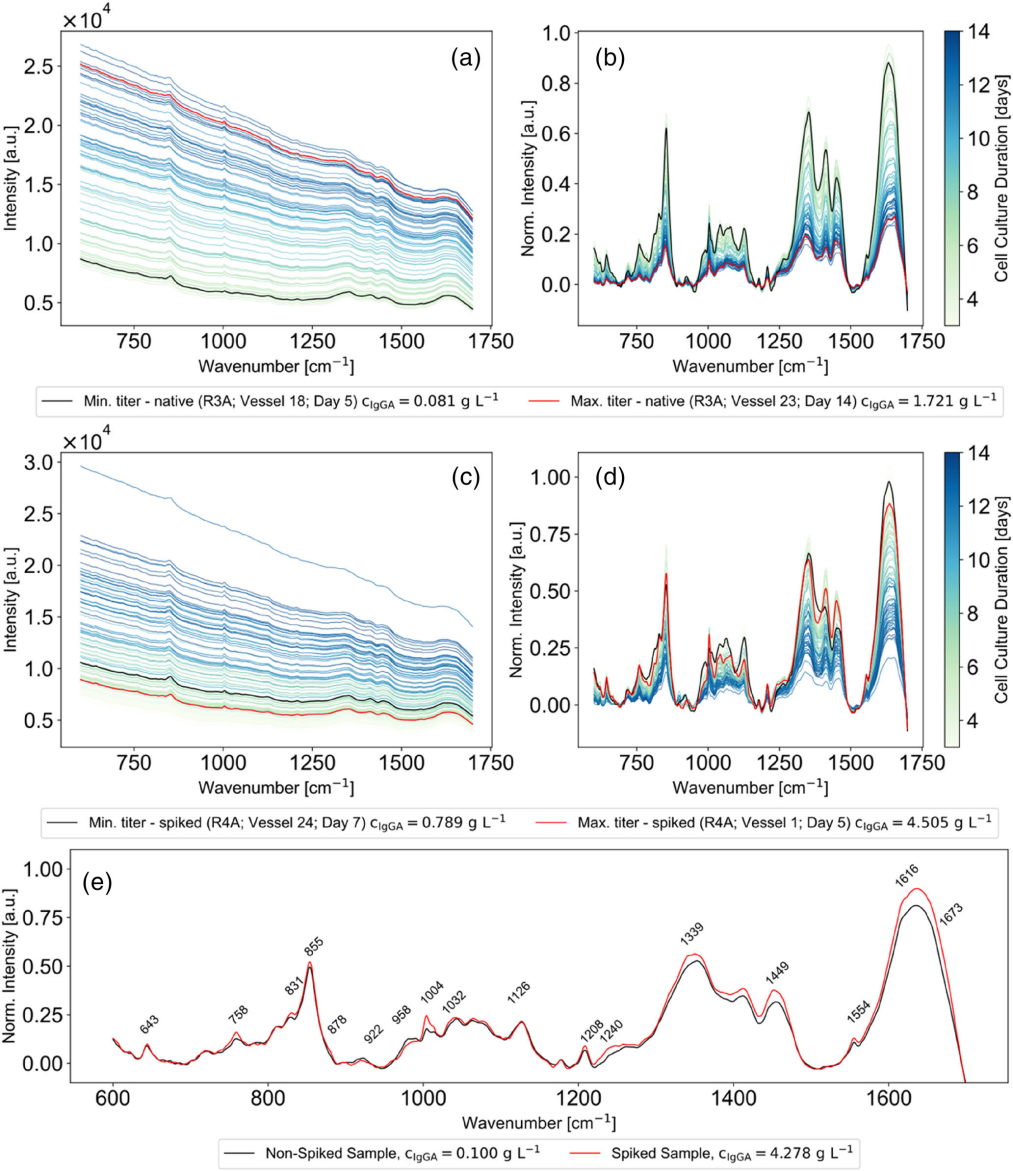
Spectral analysis of native and spiked spectra. Raw (a) and pre‐processed (b) spectra of native samples* is shown, where samples with minimal and maximal concentration of cell line A expressed IgG antibody (*c*_IgGA_) are highlighted in black and red, respectively. Similarly, raw (c) and pre‐processed (d) spectra of spiked dataset are shown and maximal and minimal c_IgGA_ spectra are highlighted. Comparison of spiked vs. native spectra for a single vessel/timepoint (R#3, vessel Nr. 20 on day 3) is shown (e) with wavenumbers highlighted based on a reference spectrum of the spiked antibody solution. *Only R3A data shown for native dataset (a, b) to ensure readability of the plot.

When investigating the raw (Figure 3a) and pre‐processed spectra (Figure 3b) of native cell culture samples, it is apparent that the spectra are dominated by increasing baseline, which correlates with cell culture progression. The increasing baseline can be attributed to an increase in autofluorescence, which has been observed across various cell culture processes.^32^ Furthermore, cell culture progression and associated autofluorescence seem to positively correlate with antibody titer (low overall intensity of minimal titer sample (c_IgGA_ = 0.081 g L^−1^), high overall intensity of sample with maximal titer (c_IgGA_ = 1.721 g L^−1^)) in raw data, with a negative correlation observed in pre‐processed data.

In contrast, the contribution of the spiked antibody signal is much lower compared to the total signal (Figure 3c (raw), Figure 3d (processed)), as evidenced by similar overall intensity for minimal (*c*_IgGA_ = 0.789 g L^−1^) and maximal (*c*_IgGA_ = 4.505 g L^−1^) antibody titer sample spectra. This is likely due to the complex spectral signal structure, since it is composed of the many overlapping spectral components of the cell culture as well as the contribution of the baseline changes. For this reason, the effect of spiking had to be visualized at a level of a single sample. Shown in Figure 3e is the effect of spiking for run R3A, vessel Nr. 20 on day 3 showing the spectra for spiked (c_IgGA_ = 4.278 g L^−1^) and native (c_IgGA_ = 0.100 g L^−1^) samples. Specific wavenumbers were highlighted where spectral differences are expected based on the reference spectra of the purified antibody (Figure S3). While for wavenumbers with a strong antibody signal the effect of spiking can be observed (such as for 758, 831, 855, 878, 1004, 1208, 1240, 1339, 1449, 1554, 1673 cm^−1^), wavenumbers with a weaker antibody signal do not show an increase in intensity as a result of spiking (643, 922, 958, 1032, 1126). Additionally, there seems to be a broad increase in intensity in the region between 1300 and 1500 and around 1650 cm^−1^ even outside of the specific antibody peaks.

Overall, while the measured dataset had a complex spectral signature as a result of containing many components with overlapping spectral signatures as well as a dynamically changing baseline, the effects of spiking were identifiable on a per sample basis, which allowed us to proceed with fitting PLS models to the data.

### 3.3 Model performance of native (non‐spiked) model

Models were calibrated for antibody titer as well as glucose and lactate. For each of the three analytes, the pre‐processing optimization routine was performed independently, and the optimal pre‐processing filter as well as model complexity (number of LVs) was selected (Table 3).

**FIGURE 4:**
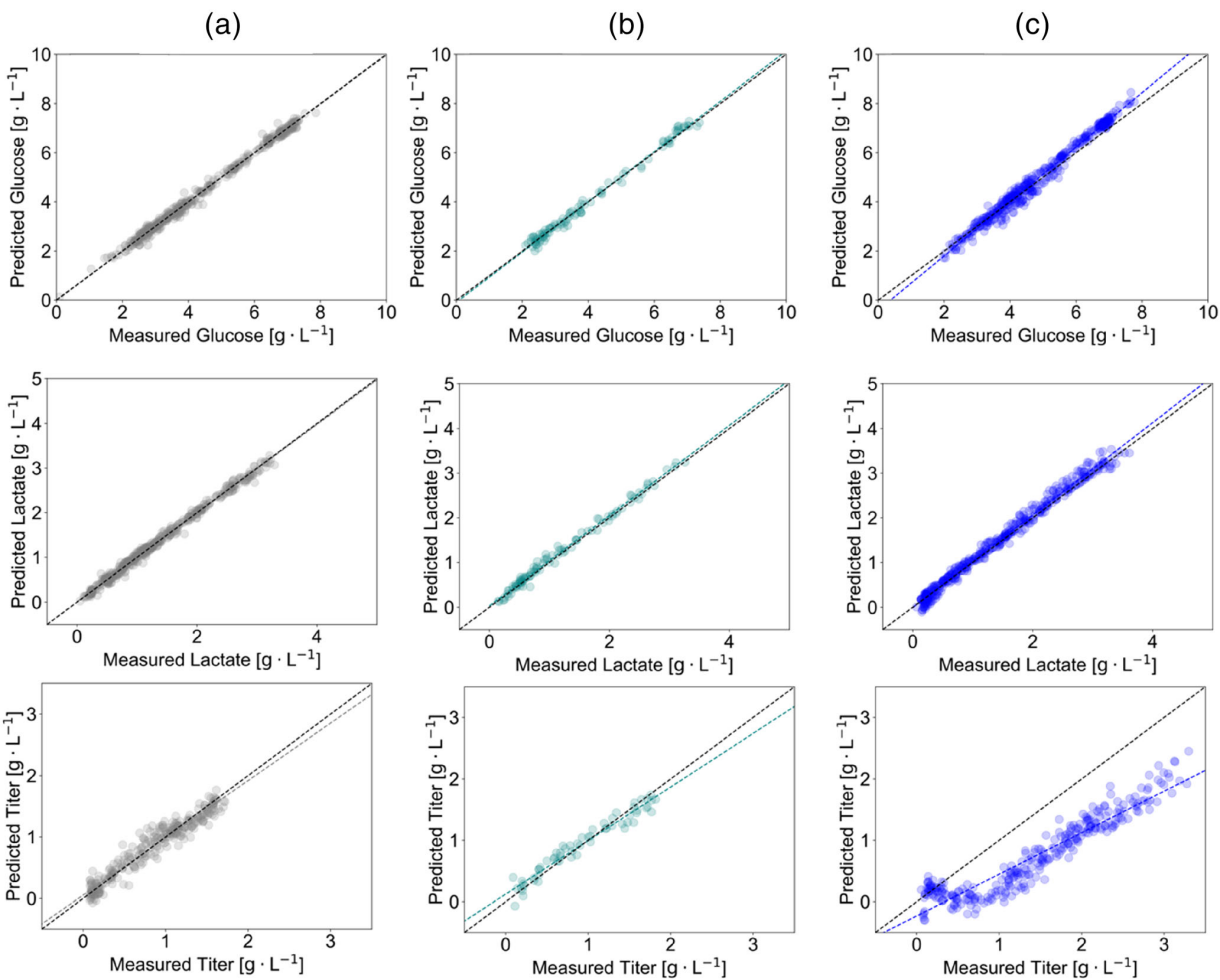
Observed versus predicted values for models calibrated using native datasets in cross‐validation (column A), test set of cell line A (column B), and cell line B (column C) for glucose (top panel), lactate (middle panel), and product titer (bottom panel).

Both the glucose (Figure 4—top panel) and the lactate models (Figure 4—middle panel) achieved good performance both in cross‐validation (nRMSECV ≤2.00%), test set of cell line A (nRMSEP of 3.00% for glucose and 2.81% for lactate), and in cell line B (nRMSEP of 4.74% for glucose, 3.32% for lactate), showing that calibration models for these two analytes could be transferred between the two cell lines tested.

The same was not true for antibody titer (Figure 4—bottom panel), where the optimized model achieved good performance in cross‐validation (RMSECV = 0.127 g L^−1^, nRMSECV = 7.76%) and on test data from cell line A (RMSEP = 0.125 g L^−1^, nRMSEP = 7.22%), but performed poorly on cell line B, where a high prediction error (RMSEP = 0.776 g L^−1^, nRMSEP = 23.93%) was observed. The observed error was due to the presence of a systematic underprediction (slope = 0.667) and non‐linearity. While the error could be explained by extrapolation, as cell line B produced higher protein titer, this seems not to be the cause of the lack of transferability as high error and underprediction were also present within the concentration range of the training cell line (0.05–1.8 g L^−1^).

### 3.4 Model performance of spiked calibration model of product titer

Next, samples spiked with the protein of interest were included in the training dataset as well as the test dataset for both cell line A and cell line B to assess if spiking can improve the titer model transferability.

**FIGURE 5:**
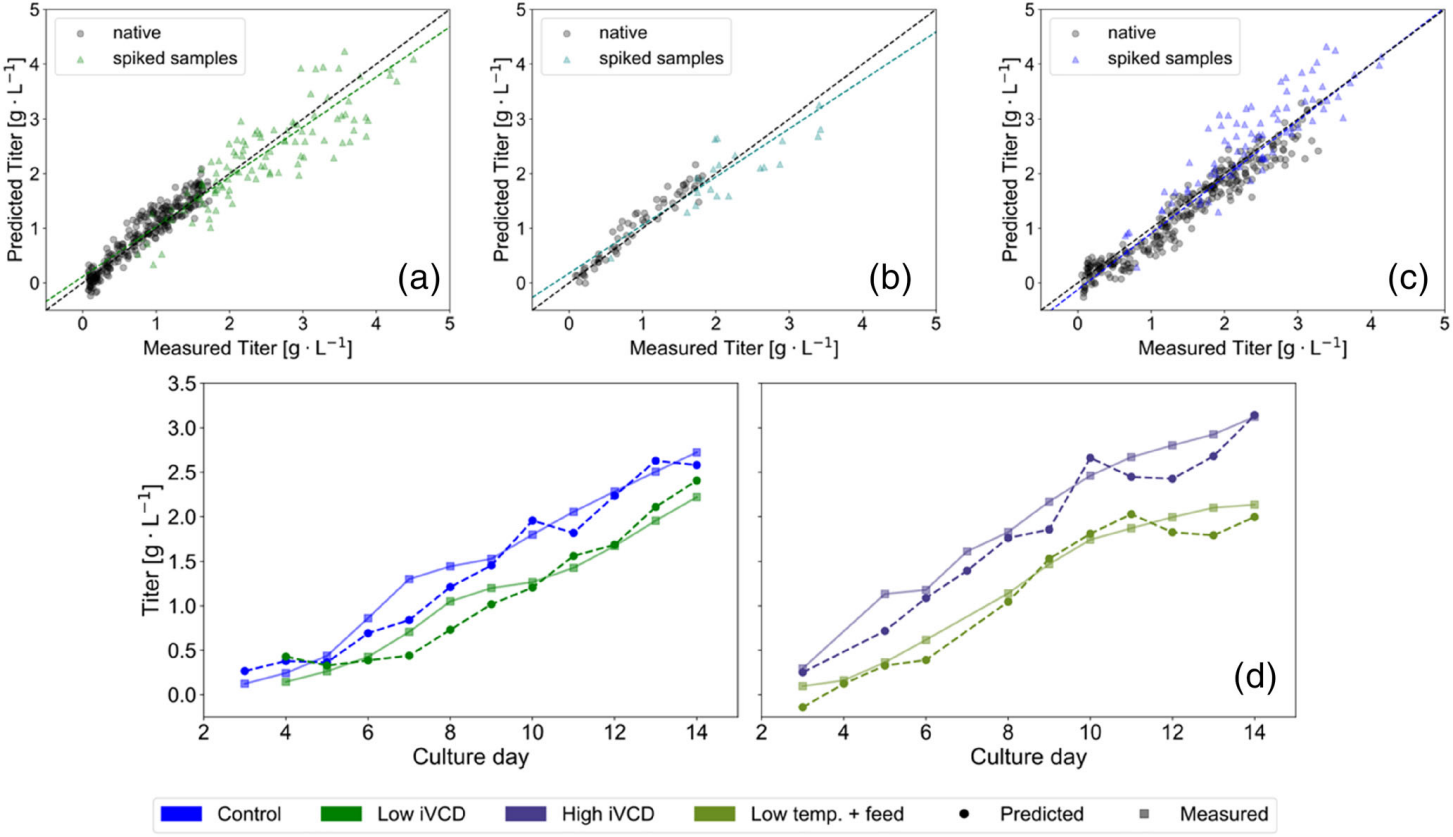
Performance of titer model calibrated using both native and spiked samples. Observed vs. predicted plots for cross‐validation (5A), test set for cell line A (5B), and test set for cell line B (5C) with predicted time‐resolved titer profiles shown across a range of conditions (5D).

While performance in cross‐validation (Figure 5a; nRMSECV of 7.60% for native titer model; 6.60% for spiked model) as well as test set for cell line A (Figure 5b; nRMSEP of 7.22% for native titer model; 7.98% for spiked model) remained comparable, major improvements were observed in the performance of the spiked model for cell line B (Figure 5c; nRMSEP of 23.93% for native titer model; 7.97% for spiked model). Examples of predicted titer profiles across a range of conditions are shown in Figure 5d. These results suggest that spiking the calibration dataset with purified antibody had a major influence on the generalization of the calibration model and resulted in a model which could be transferred across cell lines.

### 3.5 Comparison of native vs. spiked model

Important spectral features of both titer models (native and spiked) were compared to investigate the cause of poor transferability of the native model. For each model, β coefficients of the PLS were computed (including their confidence intervals generated by bootstrapping the training set). An alternative model was selected for the native calibration approach (with comparable performance to the optimal model, see Table S2) as the optimized model included pre‐processing with a derivative which would prevent direct comparison of the spectral features.

**FIGURE 6:**
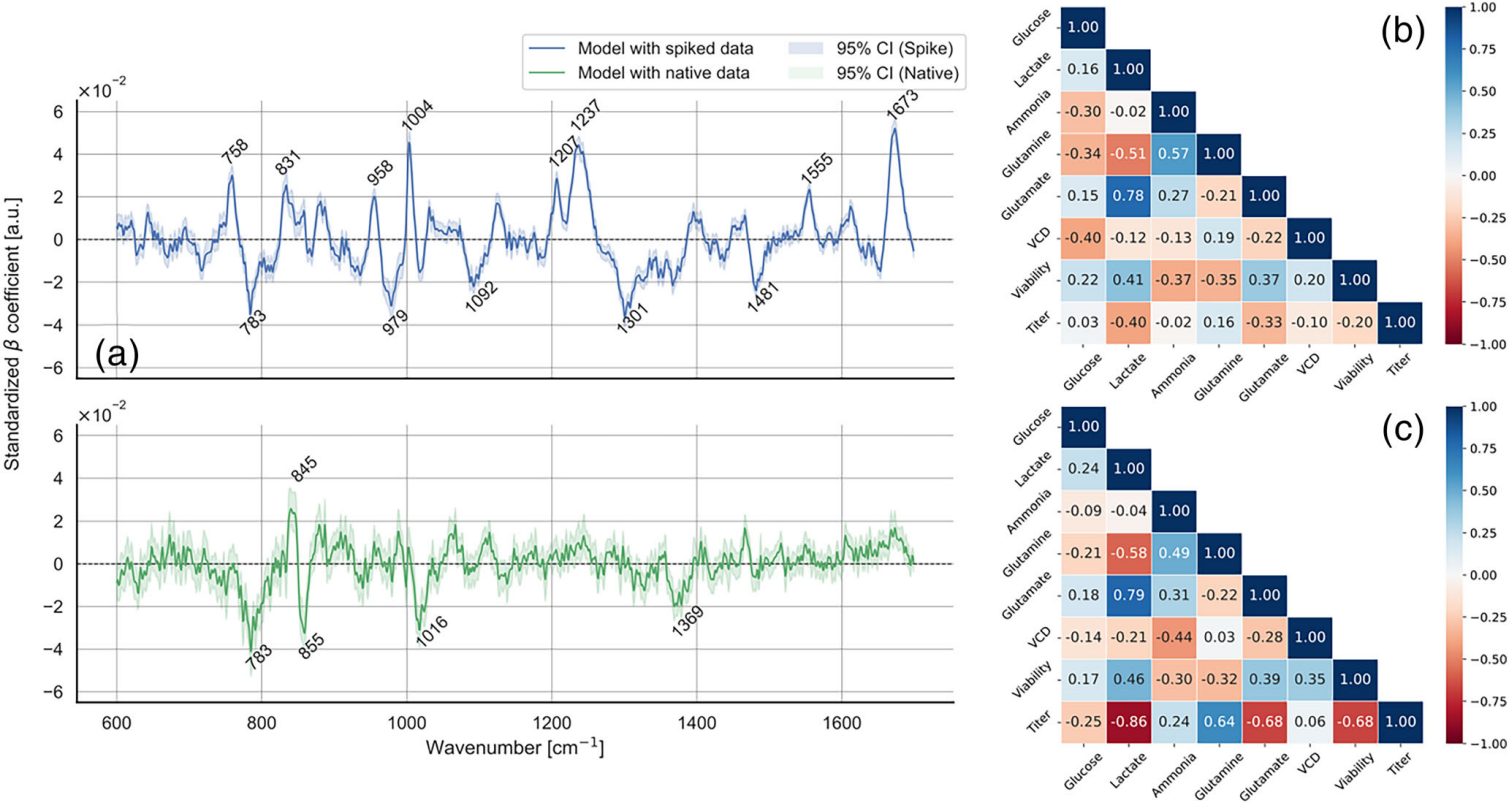
Peak assignment and analysis of the correlation structure of the native versus spiked calibration model. Beta coefficients of the PLS regression for the spiked (A, top) and native (A, bottom) are shown. Peaks with absolute *β* coefficients >0.02 are marked with their respective wavenumber. Correlation structure of the calibration datasets for spiked (B) and native (C) is shown.

When examining the resulting coefficient plots, it was apparent that the positive spectral features of the spiked model (Figure 6a—top panel) could be assigned to the peaks observed in spectra of the purified antibody (Figure S3) and as described in the literature, such as ~1004 cm^−1^ (phenylalanine), ~1237 cm^−1^ (amide III band), or ~1670 (amide I band).^43^ On the other hand, the native model (Figure 6a—bottom panel) showed no positive spectral features that could be assigned to the antibody and was dominated by negative features, such as the peak at 855 cm^−1^, which is a strong peak present in the spectra of lactate (Figure S3). Additionally, the native model showed higher noise and larger confidence intervals. Interestingly, certain features such as the peak at 783 cm^−1^ were present in both models and would require further investigation to exclude its negative effect on transfer to additional cell lines. Overall, the coefficient analysis suggested that calibrating the model using native samples only resulted in an indirect model, which was likely the cause of the observed poor transferability.

### 3.6 Estimating transferability of Raman models based on at‐line cell culture data

It is desirable to determine whether spiking is necessary prior to starting experiments, as it is resource‐intensive even with the use of high‐throughput experimentation. To this end, we investigated whether correlations of measured cell culture values can provide insights regarding the need for spiking. Pearson's correlation coefficient (*r*) was calculated using the available cell culture data across all vessels for the spiked and native datasets.

Strong negative (−0.86) correlation was observed for lactate and antibody titer in cell line A (Figure 6c). As a result of this correlation, the PLS model includes the spectral features for lactate in the model. When this model is applied to cell line B, this leads to systematic errors as such strong correlation is no longer present in cell line B (lactate‐titer *r* = −0.61, data not shown). In comparison, no measured at‐line analyte shows correlation with titer stronger than *r* = 0.40 (lactate‐titer) when spiking data are included (Figure 6b).

## 4 DISCUSSION

Monitoring the production of the target monoclonal antibody is a key capability necessary for efficient and timely decision‐making during process development and optimization. While Raman spectroscopy is a suitable PAT tool as it is able to provide near real‐time insights, generating transferable calibration models for antibody titer has previously proven to be challenging.^11^, ^12^ This study showed that utilizing a high‐throughput bioreactor system integrated with a Raman spectrometer and automated spiking enabled the development of models that are transferable across cell lines with diverse cell culture profiles, producing different monoclonal antibodies.

Models were calibrated to predict three different values—glucose and lactate concentrations and antibody titer. The former two served as controls, since transferable models were previously described.^12^, ^14^ For glucose and lactate, the (normalized) prediction errors both in the training cell line and in the new cell line were under 5% using native samples only, which was in line with the previously published reports. The titer model using native samples only showed good performance within the training cell line but resulted in large errors when applied to the new cell line. With the use of spiking, the target cell line error decreased from 24% to 8%, resulting in a much‐improved performance that is suitable for the application of titer monitoring in a process development context. The error of 8% is also within the expected range for the chosen at‐line method,^44^ where the manufacturer‐referenced error is typically within 5%, but up to 20% if the measured antibody differs in size and structure from the calibration antibody.^45^ As the calibration model inherently includes the error of the reference analytics, an improved performance could be achieved by using a HPLC based method^13^ if needed for a given application.

This work highlighted spiking as critical in disrupting the underlying correlations that are common in mammalian cell culture. In the case of the investigated training cell line, the appearance of metabolic switch (on day 5) coincided with the start of antibody production, which led to a high correlation between lactate and antibody concentration in the training set. In the new cell line (cell line B), the dynamics between lactate and antibody production shifted; specifically, the lactate switch occurred later, on day 7. This change led to inaccuracies in titer predictions since the model trained on native samples was indirect and had incorrectly linked titer to lactate production. This observation aligns with the previous report by Machleid et al., who found correlations between titer and lactate, glutamine and glutamate that degraded titer predictions across different clones.^14^ Even with high‐throughput automation, it is impractical to spike all analytes up front. As an alternative, it is advisable to investigate which correlations are present for a given cell line and associated process, then execute targeted spiking for analytes showing higher correlation. This approach relies on having a reliable panel of cell culture parameters (since confounding correlation will not be revealed if the given analyte is not measured), but with increasing accessibility of PAT, such datasets could be available as standard practice.

Additionally, it is necessary to consider how models developed using the automated high‐throughput cell culture can be transferred to other scales. As high‐throughput experimentation becomes increasingly favored across all stages of process development, obtaining reliable, near real‐time titer measurements can streamline and support the transition to manufacturing‐scale production. Reports of successful transfer between flow‐cells and in‐line probes are limited, and additional transfer between spectrometers from different manufacturers might be necessary as models are shared across various lab and production facilities, which will require dedicated pre‐processing methods.^23^

## 5 CONCLUSION

This study successfully demonstrated the development of a transferable Raman calibration model for monoclonal antibody titer across different cell lines with different metabolic profiles. By employing a high‐throughput cell culture system with automated spiking, we were able to generate a robust and transferable model in the presence of strong correlations in the native cell culture. As a result, titer monitoring can be routinely executed during process development and optimization conducted using high‐throughput cell culture, which provides additional insights that aid in decision‐making.

Our findings also highlight the importance of process knowledge in the development of Raman calibration models, illustrated in this study by the role of lactate correlation with antibody titer in degradation of predictions using native training data. The methodology presented here not only provides a framework for transferable model development but also underscores the potential of high‐throughput platforms in enhancing the efficiency and scalability of PAT model development in the biopharmaceutical industry.

## AUTHOR CONTRIBUTIONS

**Alexandra Umprecht:** methodology, software, data curation, formal analysis, writing – original draft, visualization; **Nicholas Uth:** conceptualization, methodology, investigation, writing – review & editing, supervision; **Haenah Kim:** investigation, methodology, data curation, writing – review & editing; **Oliver Spadiut:** supervision, writing – review & editing; **Yang Yang:** conceptualization, resources, writing – review & editing, supervision, funding acquisition.

## FUNDING INFORMATION

Takeda Pharmaceutical Company Limited has funded this study.

## CONFLICT OF INTEREST STATEMENT

Alexandra Umprecht is an employee of Baxalta Innovations GmbH, a member of the Takeda group of companies, and may own company stock. Haenah Kim, Nicholas Uth, and Yang Yang are currently employees or were employees of Takeda Pharmaceutical Company Limited at the time of the study and may own company stock. Nicholas Uth is an inventor on patent “Automated control of cell culture using raman spectroscopy” assigned to Lonza. Nothing to declare for the remaining authors.

## Supporting information


**Figure S1. Removal of spectra impacted by dripping –** Scatter plot of all spectra from cell line A R#1 (A), which shows spectra impacted by dripping deviating from remaining spectra along the 2nd component. (B) Examples of spectra impacted by dripping (gray), showing prominent sapphire peak, oxygen peak at 1555 cm^−1^ and missing water peak at 1645 cm^−1^.
**Figure S2. Removal of outliers of the at‐line values –** At‐line / offline values for glucose, lactate and antibody titer were investigated and outliers were removed based on prior process knowledge. Shown is an example outlier on day 13, where antibody concentration drops, but recovers on day 14. An assumption was made that this is due to an analytics error.
**Figure S3. Reference spectra of lactate (24 g L** ^**−1**^ **in DMEM media) and purified monoclonal antibody**. The spectra of antibodies have been pre‐processed using SNV normalization and AsLS baseline removal.
**Table S1. List of pre‐processing functions with selected parameter values**.
**Table S2. Comparison of optimal model for native model and a model selected for coefficient analysis**.

## Data Availability

Research data are not shared.
