# Supplementary material for: Rapid development of a transferable Raman model using high‐throughput cell culture for monitoring monoclonal antibody titer
Source: Biotechnol Prog. 2026 Apr 5;42(3):e88506. doi: 10.1002/btpr.88506 (PMC13266923; doi:10.1002/btpr.88506)
Supplement: Supplementary file 1 — Figure S1. Removal of spectra impacted by dripping – Scatter plot of all spectra from cell line A R#1 (A), which shows spectra impacted by dripping deviating from remaining spectra along the 2nd component. (B) Examples of spectra impacted by dripping (gray), showing prominent sapphire peak, oxygen peak at 1555 cm−1 and missing water peak at 1645 cm−1. Figure S2. Removal of outliers of the at‐line values – At‐line / offline values for glucose, lactate and antibody titer were investigated and outliers were removed based on prior process knowledge. Shown is an example outlier on day 13, where antibody concentration drops, but recovers on day 14. An assumption was made that this is due to an analytics error. Figure S3. Reference spectra of lactate (24 g L −1 in DMEM media) and purified monoclonal antibody. The spectra of antibodies have been pre‐processed using SNV normalization and AsLS baseline removal. Table S1. List of pre‐processing functions with selected parameter values. Table S2. Comparison of optimal model for native model and a model selected for coefficient analysis. [file BTPR-42-e88506-s001.docx]

**Supplementary Information**


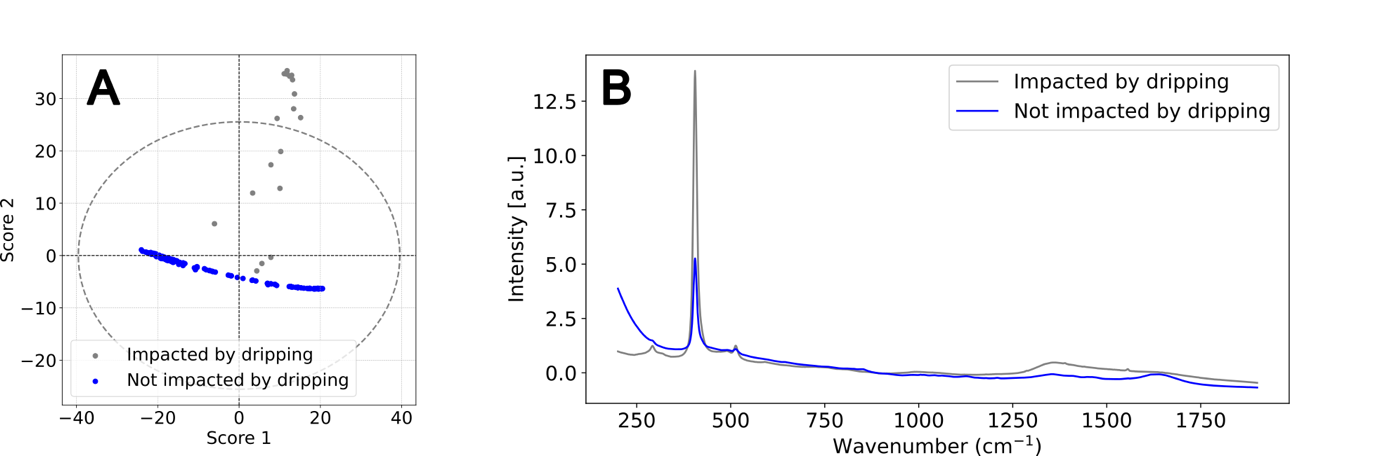


**Figure S1 Removal of spectra impacted by dripping –** Scatter plot of all spectra from cell line A R#1 (A), which shows spectra impacted by dripping deviating from remaining spectra along the 2^nd^ component. (B) Examples of spectra impacted by dripping (grey), showing prominent sapphire peak, oxygen peak at 1555 cm^-1^ and missing water peak at 1645 cm^-1^.


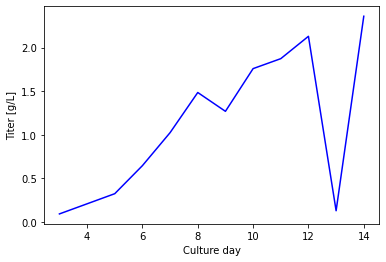


**Figure S2 Removal of outliers of the at-line values –** At-line / offline values for glucose, lactate and antibody titer were investigated and outliers were removed based on prior process knowledge. Shown is an example outlier on day 13, where antibody concentration drops, but recovers on day 14. An assumption was made that this is due to an analytics error.

**
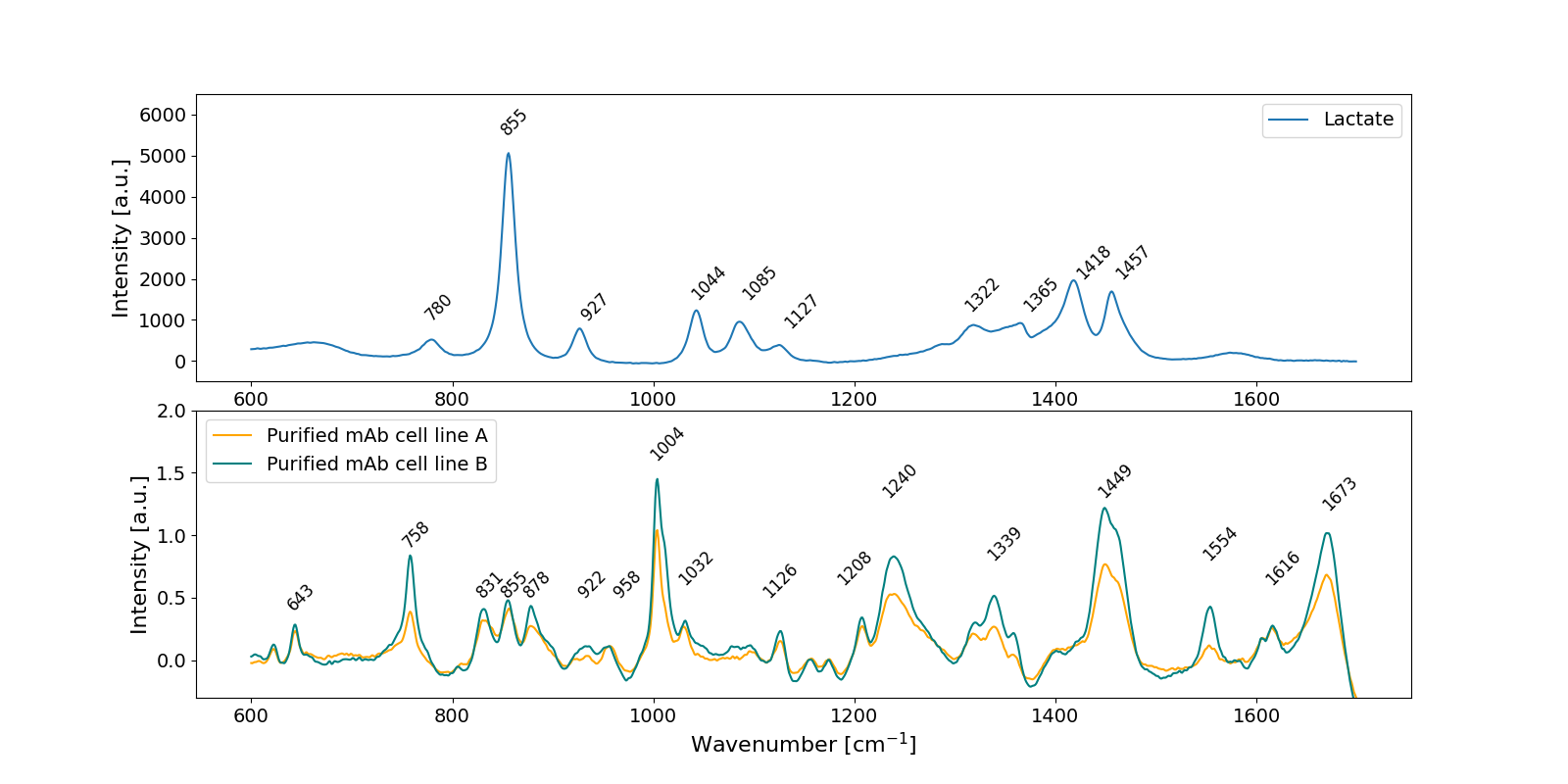
**

**Figure S3 Reference spectra of lactate (24 g L^-1^ in DMEM media) and purified monoclonal antibody.** The spectra of antibodies have been pre-processed using SNV normalization and AsLS baseline removal.

**Table S1 List of pre-processing functions with selected parameter values**

| **Pre-processing function** | **Parameter(s)** | **Values** |
| --- | --- | --- |
| Robust Normal Variate | Percentile range max; min | (75; 25), (65; 35), (85; 15) |
| Modified Polynomial | Polynomial order | 2, 3, 4 |
| Improved Modified Polynomial | Polynomial order | 2, 3, 4 |
| Asymmetric Least Squares (baseline correction) | Smoothness (λ); asymmetry (p) | (10^6^; 0.1), (10^7^; 0.1), (10^6^; 0.01), (10^7^; 0.01) |
| Adaptive Iteratively Reweighted Penalized Least Squares | Smoothness (λ) | 10^3^, 10^4^, 10^5^ |
| Savitzky-Golay (baseline correction) | Window size; derivative order; polynomial order | (5; 1; 2), (5; 2; 2), (5; 1; 3), (5; 2; 3),  (7; 1; 2), (7; 2; 2), (7; 1; 3), (7; 2; 3), (9; 1; 2), (9; 2; 2), (9; 1; 3), (9; 2; 3) |
| Savitzky-Golay (denoising) | Window size; polynomial order | (5; 2), (5; 3), (9; 2), (9; 3) |
| Asymmetric Least Squares (denoising) | Smoothness (λ); asymmetry (p) | (10; 0.01), (50; 0.01), (100; 0.01),  (10; 0.1), (50; 0.1), (100; 0.1) |

**Table S2 Comparison of optimal model for native model and a model selected for coefficient analysis**

|  | **Optimal model** | **Model used for ß coefficient comparison** |
| --- | --- | --- |
|  | **Training set (cell line A)** |  |
| **No. of Samples (n)** | 275 | 275 |
| **Pre-processing** | SNV; S-G (1^st^ der); AsLS | SNV; AsLS; S-G |
| **LVs** | 6 | 6 |
| **(n)RMSECV [g L^-1^; %]** | 0.127; 7.759 | 0.139; 8.488 |
| **R^2^** | 0.932 | 0.919 |
| **Bias [g L^-1^]** | 0.052 | 0.063 |
| **Slope** | 0.932 | 0.925 |
|  | **Test set (cell line A)** |  |
| **No. of Samples (n)** | 60 | 60 |
| **(n)RMSEP [g L^-1^; %]** | 0.125; 7.221 | 0.123; 7.140 |
| **R^2^** | 0.953 | 0.956 |
| **Bias [g L^-1^]** | 0.123 | 0.114 |
| **Slope** | 0.873 | 0.868 |
|  | **Test set (cell line B)** |  |
| **No. of Samples (n)** | 290 | 290 |
| **(n)RMSEP [g L^-1^; %]** | 0.776; 23.934 | 0.659; 20.330 |
| **R^2^** | 0.883 | 0.895 |
| **Bias [g L^-1^]** | -0.230 | -0.088 |
| **Slope** | 0.667 | 0.670 |
